# Safety-Constrained Agentic AI for Autism Screening: A Multimodal, Clinician-Guided Architecture

**DOI:** 10.7759/cureus.108961

**Published:** 2026-05-16

**Authors:** Debashis Patra, Ambar N Saha, Som S Mukherjee

**Affiliations:** 1 Artificial Intelligence and Machine Learning, The University of Texas at Austin, Woodstock, USA; 2 Information Technology/Software Engineering, Cognizant Technology Solution, Halifax, CAN; 3 Information Technology/Software Engineering, Projee, Inc, Woodstock, USA

**Keywords:** agentic ai, ai governance, autism screening, autism spectrum disorder (asd), clinical decision support, early screening, healthcare technology, multimodal machine learning

## Abstract

Autism spectrum disorder (ASD) diagnosis often encounters substantial delays due to several reasons, such as shortages of trained specialists and limited access to care in rural and underserved communities. Moreover, it is very difficult to perform behavioral assessments within a single clinical visit, as it is significantly dependent on the child's behavior. Delayed diagnosis can postpone early intervention, which is important for improving developmental outcomes in children with ASD. Although artificial intelligence (AI) is increasingly explored in healthcare, its adoption in ASD screening remains limited due to concerns about reliability, governance, consent management, bias, and clinical trust.

In this work, we propose a conceptual, governance-driven, clinician-augmented AI framework designed to assist clinicians during the ASD screening process rather than replace them. The proposed architecture collects various inputs such as text, audio, and video of a child from parents, schools, or caregivers, and then it runs through multiple specialized agents who are responsible for consent validation, bias monitoring, model selection, confidence-based abstention, and providing a structured report which will help clinicians in their assessment. Caregivers receive only non-diagnostic guidance, while clinicians receive structured decision-support information designed to aid clinical evaluation.

The main goal of this article is not to validate model performance. We are mainly trying to design an agentic framework where governance and safety rules can be managed properly through multiple specialized agents. Although we have performed a single model training using a ResNet-50 facial-image classification model on a publicly available dataset, our main goal was to validate the governance and multi-agent system. The Stage 1 governance validation was done using more than a hundred scenarios.

It is very important to highlight that our article should be viewed as a conceptual governance-driven agentic framework with Stage 1 validation, and it is definitely not a fully workable clinical solution. In the next phases, we plan to collect clinically validated data and focus more on model training, multimodal integration, and real-world validation.

## Introduction

It is broadly accepted that early detection of autism spectrum disorder (ASD) is critical because early intervention shows promising outcomes in the social development of impacted children [1,2]. Unfortunately, even in the most developed countries of the world, early detection of ASD using available diagnostic approaches continues to be a challenge [3,4]. It is paradoxical that, even in the age of artificial intelligence (AI), most ASD screenings are still conducted by clinicians during in-person sessions [5]. Although this manual screening represents the standard of care for the detection of ASD, it has inherent disadvantages that are difficult to overlook. First, due to the lack of expertise and resources, there is often a long wait time before a child receives their first screening session. Even when family members directly observe signs of autistic behavior in the child, they are unable to seek immediate early intervention before the screening. Second, it is very difficult to accurately detect ASD based on a single in-person session because the behavior of the child can vary depending on their mental state on the day of screening [6].

Prior studies demonstrate promising results, but those are focused mostly on controlled evaluation settings [7,8]. While these processes were robust methodologically, they did not provide a well-defined strategy for broad adoption, enabling real-world users to apply the techniques with a high degree of confidence. In many cases, the discussion focused on how these models performed across diverse demographics or populations not included in the training data [8].

The recognition of this knowledge gap prompted us to propose a reliable ASD screening framework that could be adopted sustainably in real-world diagnostic settings, leading to more accurate diagnoses and an accelerated diagnostic cycle [9,10]. We considered a diverse set of variables, especially focusing on real-world situations where an in-person diagnosis would not be accurate/reliable, and the resulting impact on children and families.

Conceptually, the proposed system follows a structured flow where input data are processed by AI models, followed by governance checks (e.g., consent validation, bias monitoring, and confidence evaluation), before being presented as decision support or escalated to clinicians.

The primary objective of this study is to propose a governance-driven, clinician-augmented AI framework for ASD screening and to evaluate its governance behavior under controlled Stage 1 validation conditions using synthetic scenarios and a preliminary model benchmark.

## Materials and methods

Related work

Prior research on ASD screening using AI can be broadly categorized into three areas.

First, a significant amount of research focuses on developing a proper AI model for ASD detection and evaluating its performance under controlled environments [7-11]. Although the reports are very promising, they are limited to curated datasets, and their applicability in real-world diagnostics remains uncertain.

Second, several studies have explored model performance across diverse populations and demographic groups [8]. While these efforts improve understanding of model robustness, they often do not integrate these findings into deployable clinical workflows.

Third, emerging discussions highlight the importance of safety, fairness, and interpretability in medical AI systems, including aspects such as consent handling, bias monitoring, and uncertainty-aware decision-making [12-20]. However, these governance-related mechanisms are often underreported or not explicitly operationalized within end-to-end system designs.

In contrast, our work focuses on bridging this gap by proposing a governance-driven, clinician-augmented AI agentic framework that explicitly incorporates consent validation, bias monitoring, and confidence-based abstention within a structured system architecture designed for real-world deployment.

System architecture

**Figure 1:**
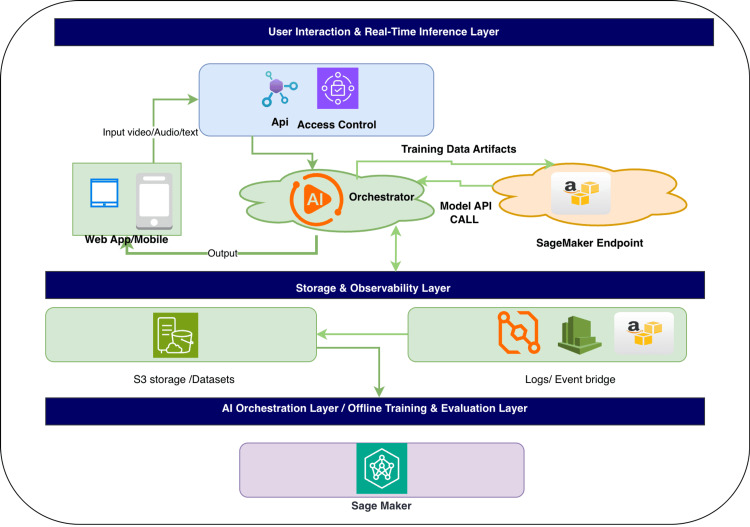
Architecture Layer Architecture of the agentic AI system showing interaction between the user interface, AI orchestration layer, agent-based inference, cloud-based machine learning services, and storage/observability components. Image credit: The authors, using draw.io (diagrams.net).

Our proposed architecture consists of five logical layers, where each layer has a clear and separate responsibility [12-16]. We have designed it in such a way that maintainability will be effortless, easy to understand, and everything can be audited [17-20]. Instead of combining everything into one place, each layer is given a clear responsibility. This separation helps in keeping important governance checks like consent validation, bias monitoring, and confidence evaluation visible and testable [21-23]. It also ensures that the system does not learn unintentionally during runtime and that every output can be traced back to the exact data, model, and decisions that produced it [24,25]. Threshold values are treated as configurable parameters and will be calibrated in future work using real-world data (Figure 1).

The user interaction layer is our API layer, which is the entry point where caregivers and clinicians interact with the system through web or mobile applications. Caregivers or parents provide inputs, such as text, audio, video, and behavioral observations, while clinicians receive structured reports that will help them make the final decision. This layer focuses on usability for non-technical users while maintaining secure access and proper role-based communication.

The agent orchestration layer acts as the central controller of the system. It coordinates multiple specialized agents responsible for tasks such as consent verification, bias and applicability checks, model selection, confidence evaluation, and final report generation. Instead of embedding all this logic into application code, we keep it as separate responsibilities so that every decision remains transparent and easy to audit. This layer also ensures that the system produces output only when all required conditions are satisfied and can abstain when confidence is low, which is critical to build trust in clinical scenarios.

The storage and observability layer manages all system data and tracking. It stores user inputs, processed data, embeddings, model artifacts, generated outputs, and detailed logs of every step. Observability ensures that every action taken by the system can be traced later if needed. This layer plays an important role in preserving transparency, data lineage, and compliance. It also supports a fail-safe behavior (see Appendix J). For example, if input validation is incomplete, logs are missing, or model traceability cannot be verified, the system abstains from producing a result and escalates the case for human review. This ensures that no decision is made without proper validation, auditability, and oversight.

The real-time inference layer is where predictions are generated. It uses only pre-approved and validated machine learning models, and these models are invoked only after all governance checks from the orchestration layer are completed. No training or modification happens at this stage, which ensures that outputs stay consistent, stable, and reliable.

The offline training and evaluation layer is responsible for building and improving models in a controlled environment. It includes data preparation, model training, validation, bias checks, and version management through a model registry. Only models that pass all validation and governance checks are promoted to the real-time system. This separation between training and inference ensures that experimental changes never directly impact live predictions.

**Figure 2:**
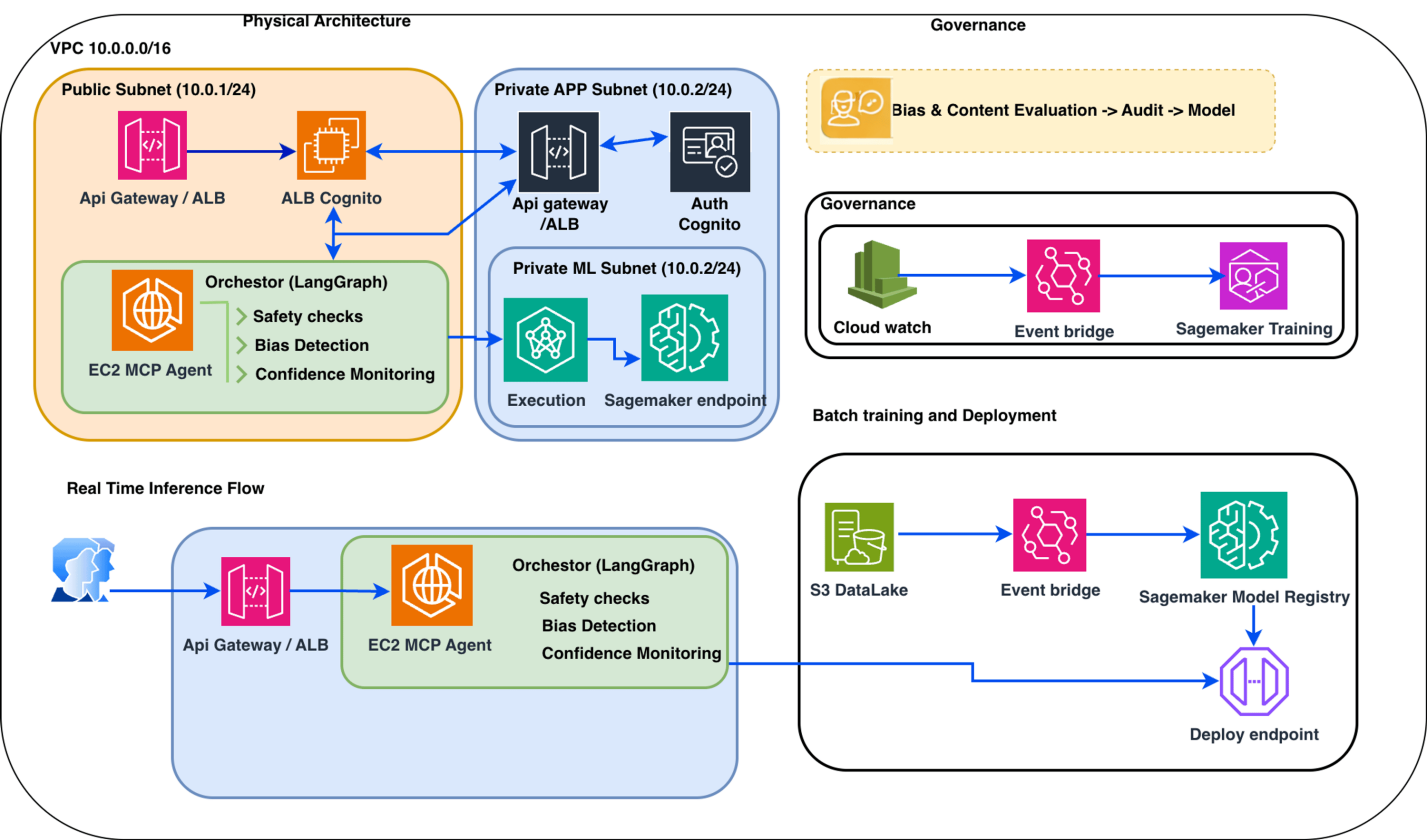
AI Agents' Workflow in AWS Environment Deployment of AI agents inside an AWS VPC. Caregivers and clinicians authenticate via Amazon Cognito; requests flow through an API gateway/ALB and SQS into a LangGraph-based agent system that performs safety, confidence, and planning checks, integrates with an Amazon S3 Vectors retrieval layer, and issues real-time predictions through SageMaker endpoints. Image credit: The authors, using draw.io (diagrams.net).

Figure 2 shows how our proposed system fits into the AWS architecture (Amazon Web Services, Inc., Seattle, WA, USA). The system handles both real-time requests and model training in a separate way.

User requests first come through API Gateway and load balancers, where authentication is handled by Cognito (AWS Authentication Service). From there, the request goes to the central orchestration (LangGraph, LangChain Inc., San Francisco, CA, USA; see Appendix C), which performs safety checks, bias detection (see Appendix D), and confidence validation. Once those checks are passed, the request is sent to the SageMaker (Amazon Web Services, Inc., Seattle, WA, USA) endpoint for inference, and the result is returned to the user if the model confidence is above the threshold.

All activities are logged using CloudWatch (Amazon Web Services, Inc., Seattle, WA, USA) and EventBridge (Amazon Web Services, Inc., Seattle, WA, USA) for monitoring and audit purposes. If any critical check fails, then the system should stop the process and return an error message to the user, and also log the scenario properly.

On the training side, if the system receives consent to use the user's data for training purposes, then EventBridge triggers the training pipeline, and data is retrieved from the S3 bucket, which has already been stored as part of the user data submission. Only approved models are registered and deployed to SageMaker endpoints. This ensures that real-time inference always uses validated models.

Overall, the architecture keeps real-time inference, training, and monitoring separate but connected, so the system remains secure, controlled, and reliable.

Agent orchestration and workflow

In this section, we are going to discuss the importance of introducing multiple agents and the workflow between those agents. Intentionally, we have proposed a distributed architecture where each agent will handle an important and distinct responsibility [12-16,21-23], which will make the entire system more scalable, easy to understand and maintain, easy to implement proper auditing, and can be improved without hampering the entire structure of the system.

Consent and Ethics Agent

This is the first agent that gets invoked when a parent or caregiver uploads text, audio, video, images, or behavior notes. This agent verifies whether proper consent is given and whether the requested processing is permitted. If those conditions are not satisfied, then the agent stops the workflow immediately.

Bias and Applicability Agent

The responsibility of this agent is to check whether the incoming data is suitable for inference or not. It checks missing inputs, weak quality, unusual conditions, or, in some cases, where the model is not equipped to perform reliably. For example, the input data has come from a different demography, which was not present during model training. In such scenarios, the system can pause the workflow, request better input, or abstain from moving forward.

Model Selection and Execution Agent

This model inspects the input data and chooses the most appropriate model from the secure model registry to perform inference. All the models that are in the model registry are approved models, all have passed offline testing, and those were added to the registry after formal approval.

Confidence and Abstention Agent

The prediction is then reviewed by the Confidence and Abstention Agent. This agent checks whether the result is strong and consistent enough to be trusted. If confidence is low (see Appendix F) or signals are conflicting, the system can abstain instead of returning an unreliable output [17-20,25].

Reporting Agent

Finally, the Reporting Agent prepares the final response. Parents or caregivers receive simple non-diagnostic summaries, while clinicians receive a more detailed structured report with observations, confidence indicators, and supporting context.

Throughout the workflow, logs are maintained for traceability and audit purposes. If any critical governance check fails, the system avoids generating output. In healthcare scenarios, safety is more important than speed. (See Appendix I for a detailed-level flowchart and algorithm.)

Evaluation and Validation Approach

Right now, our proposed system is still in Stage 1 validation (see Appendix G), as it needs to be validated very thoroughly in a controlled environment before it can be used in real-world settings. First, we should check whether the governance rules, safety measures, and agent workflows are working as expected under a controlled environment, and at this stage, our goal is not live deployment. Before checking the model performance, we need to verify all the important features, like inference should not proceed without valid consent, and also the system should not provide any output if it detects applicability risk or if the input is weak in quality.

**Figure 3:**
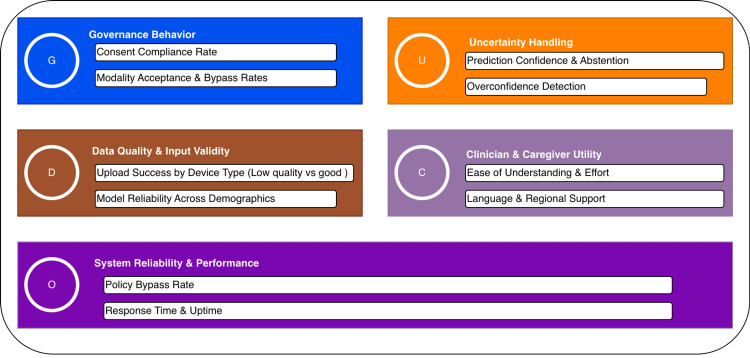
Proposed Evaluation Metrics Evaluation dimensions covering governance behavior, data quality and applicability, uncertainty handling, clinician and caregiver utility, and operational reliability. Image credit: The authors, using draw.io (diagrams.net).

Model accuracy alone is not the driving factor [17-20] in this system. As shown in Figure 3, it is also important to see how the system behaves in practice, which includes whether governance rules are actually followed, whether uncertainty is handled safely, whether input quality is acceptable, and whether clinicians and caregivers find the system useful. Reliability during operation is also an important part of this evaluation.

**Figure 4:**
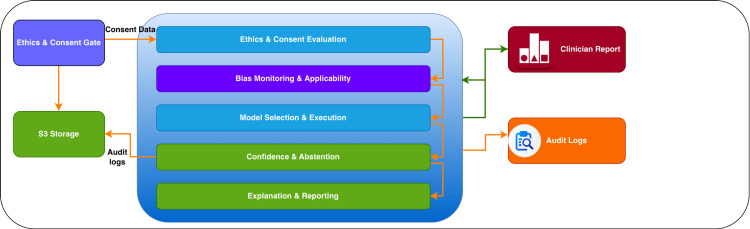
Multi-Agent Workflow for Clinician-Augmented Autism Screening Agents validate ethical and consent requirements for personal health information (images, video, audio). Data are not used for training without explicit consent. All security events are logged to S3 and used for anomaly detection. Image credit: The authors, using draw.io (diagrams.net).

Figure 4 depicts how the multi-agent system works together in practice. It begins with consent validation, and once the consent is properly given and verified, the system stores the input data and proceeds to the next step, which is Bias Monitoring and Applicability. After that, the agent selects the proper model and checks whether to abstain from providing any output if the confidence score is low. Finally, it generates the report, and it is mandatory to maintain logs so that the decisions can be traced if needed.

We have taken the safety measures very seriously because our goal is to make this system work in real-world settings. Table 1 highlights all the common failure scenarios that may occur during execution. We have consolidated all the possible failure scenarios, the severity of those errors, and the mitigation plans. Each scenario is tied to a specific agent so that fixes can be applied to one agent’s workflow while keeping other agents’ behavior intact.

**Table 1:** Failure Mode and Hazard Analysis

Failure Mode	Responsible Agent	Severity	Potential Effect	Mitigation
Consent is missing at report generation	Ethics & Consent Evaluation Agent	High	Invalid consent; privacy violation	Pre-generation consent gate; pipeline halts on failure
Low-quality input is causing quality gaps	Confidence & Abstention Agent	Medium	Garbage-in, garbage-out predictions	Input-quality thresholds; abstention triggered
Unvalidated model used at inference	Model Selection & Execution Agent	High	Inference on underrepresented populations	Signed registry gate; deployment-approval required
Wrong language/tone to the caregiver	Explanation & Reporting Agent	Medium	Confusion or misleading guidance	Role-based templates; vocabulary filter
Model artifact tampering or drift	Model Selection & Execution Agent	Critical	Unmonitored malfunction	Cryptographic signing; registry integrity checks
Audit log write failure	Audit & Logging Agent	High	No traceability for decisions	Fail-closed: output suppressed; alert on any failure
Audit log clock/ordering skew	Audit & Logging Agent	High	Unreliable reconstruction of episodes	Time-sync monitoring; log-ordering alerts

We should choose a gradual rollout instead of directly jumping into large-scale deployment. Once the Stage 1 evaluations are successful, we need to perform synthetic data validation, clinician-supervised studies, caregiver testing and feedback, and finally, a proper evaluation across different demographic groups.

Model, data, and source code

All the image data used for training and testing were collected from publicly available autism-related datasets on Kaggle (datasets/ronakp004/autism-spectrum-detection-from-kaggle-zenodo) [26]. The dataset is roughly 5 GB and mainly contains facial images from different angles to capture certain behavioral signs. The model is built on a ResNet-50 architecture (Microsoft Research, Redmond, WA, USA) (around 25M parameters). The trained weights are about 100 MB, while the full set of training artifacts comes to around 10 GB. (See Appendix E regarding training details.)

We have maintained a balance between autism and non-autism classes based on the data we collected. The demographic information in the dataset is somewhat limited and not fully consistent, but it does include critical attributes such as age, gender, ethnicity, geographic origin, and social background. There is also natural variation in the dataset-image quality, pose, orientation, behavioral expression, and how the data was captured. This variation helps the model become more robust, but at the same time, it introduces some noise into the learning process.

The data is divided into three parts: the first set is used for training the model, the second dataset is used for tuning the model, and the final set is used for testing. It is very important to note that this image-based model represents a single modality, and we have used this data to demonstrate our proposed framework. It is not sufficient for clinical use. We need to train multiple models using larger and more diverse data types, such as videos, images, text, and audio, before they can be deployed in real-world settings.

The dataset source is available in Ref. [26], and the source code in Ref. [27].

## Results

Scenario testing

We have prepared more than a hundred governance-focused test suites, and the framework was evaluated under controlled Stage 1 conditions. We mainly focused on the following governance validations -- consent violations, unauthorized requests, policy conflicts, abstention situations, noisy or incomplete inputs, escalation flows, and model approval restrictions. For simplicity, only a few representative governance scenarios are discussed in this paper to show important decision outcomes such as Deny, Abstain, and Proceed. As part of the Stage 1 evaluation phase, we were able to confirm that our system is able to follow all the governance rules under a controlled environment. For example, the system denies requests if consent is not given explicitly, and the system abstains from providing any output if it finds any uncertainty or conflict in the output, etc.

Moreover, we checked a couple of important scenarios other than governance rules during the testing phase, such as caregivers receiving only non-diagnostic reports, unauthorized model promotion or registry modification being restricted, and all audit-related information being properly captured during each phase of the entire workflow, etc (Table 2).

**Table 2:** Synthetic Test Cases and Observed Decisions SNR, signal-to-noise ratio.

Scenario ID	Scenario Name	Input Condition	Expected Action	Decision	Signal
S-01	Consent Absent	No consent provided	Block pipeline	Deny	Pipeline Halted
S-02	Low-SNR Audio	Noisy audio input	Disable modality	Abstain	Modality Disabled
S-03	Converging Signals	All signals agree	Allow execution	Proceed	None
S-04	Conflicting Signals	Signals conflict	Trigger abstain	Abstain	Conflict Detected
S-05	Unauthorized Request	Caregiver asks for clinician data	Deny access	Deny	Access Denied
S-06	Policy Denied	Violates policy	Enforce restriction	Deny	Access Denied
S-07	Repeated Abstentions	Multiple uncertain cases	Escalate	Abstain	Escalation
S-08	Untrusted Model	Model not approved	Reject model	Deny	Model Rejected
S-09	Caregiver Language	Non-clinical required	Filter language	Proceed	Vocab Filter
S-10	Consent Change	Sensitive/updated consent	Restrict data	Abstain	Modality Excluded

At the same time, it is important to mention that in our Stage 1, we have concentrated on validating the governance behavior in the entire screening workflow, and in future phases, we will focus on proper model training with diverse datasets, perform broader stress testing, and gradually make it ready for real-world use. See Appendix H for more details on test cases.

Model performance

It is crucial to note that the reported model performance metrics are included primarily as preliminary proof-of-concept outputs, while the main objective of this work is to evaluate the behavior and governance feasibility of the proposed agentic AI architecture under controlled Stage 1 conditions rather than to establish clinically validated predictive performance.

**Figure 5:**
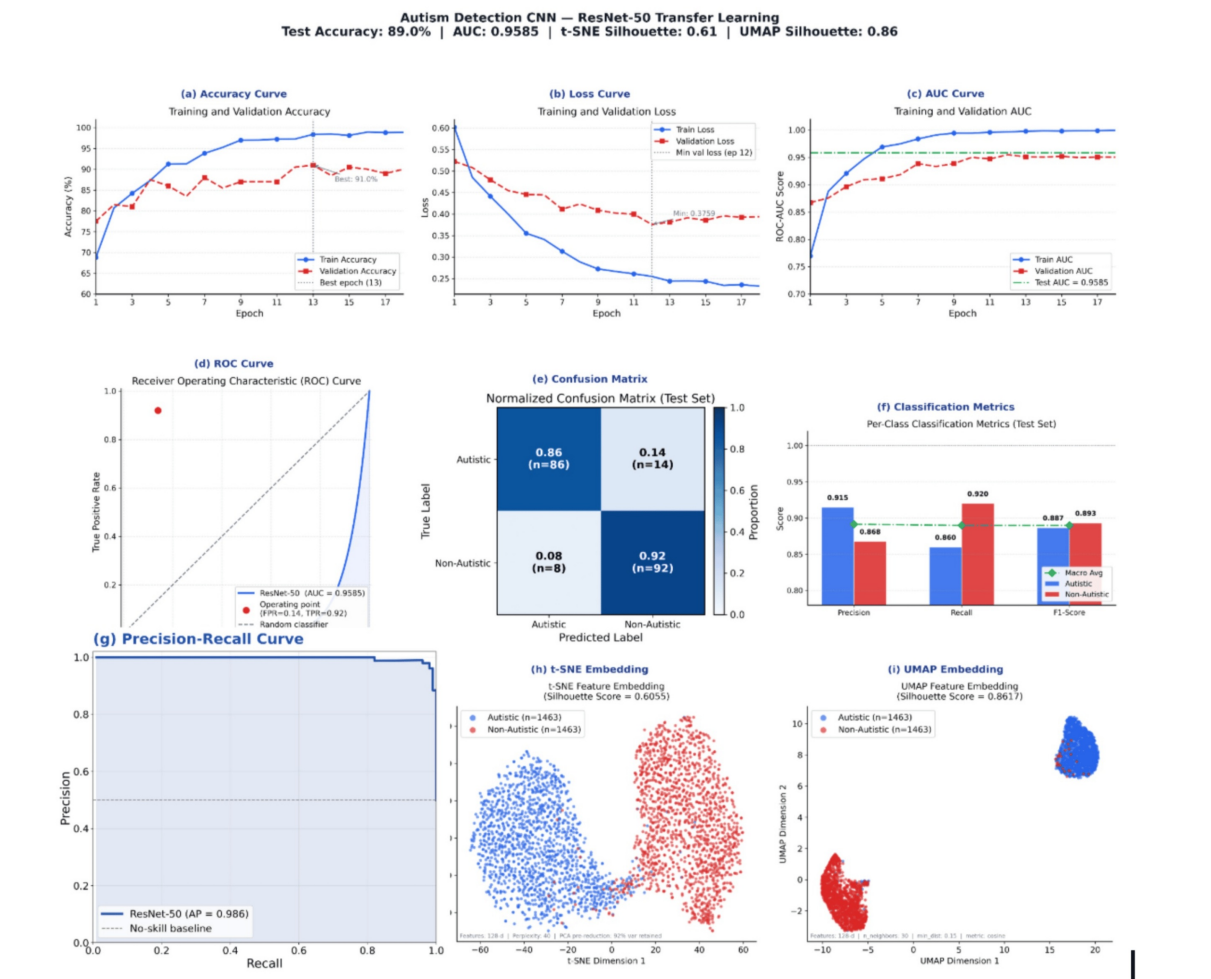
Graphs Showing the Performance Evaluation of ResNet-50 for Autism Screening (a) Accuracy curve shows how training (blue) and validation (red) accuracy change across epochs. The best validation accuracy, around 91.0%, is achieved near epoch 13. (b) Loss curve shows training (blue curve) and validation (red curve) loss. The lowest validation loss is around 0.375, which is seen near epoch 12. (c) AUC curve shows training (blue curve) and validation (red curve) AUC over epochs. The final test AUC is around 0.9585 (shown as the green dashed line). (d) ROC curve on the test set gives an AUC of 0.9585. The red point shows the selected operating threshold (false positive rate ≈ 0.14, true positive rate ≈ 0.92). (e) Normalized confusion matrix (test set) (rows = actual labels, columns = predicted labels): 0.86 (n = 86) of autistic cases are correctly classified. 0.14 (n = 14) of autistic cases are classified as non-autistic. 0.08 (n = 8) of non-autistic cases are classified as autistic. (f) Confidence and Abstention Agent workflow. (g) Bias Monitoring and Applicability Agent workflow. (h) Explanation and Reporting Agent workflow. (i) Audit Logging and Governance Traceability workflow. ROC, receiver operating characteristic; AUC, area under the curve.

Here is the result of the ResNet-50 model using a held-out test dataset (Table 3, Figure 5). 

**Table 3:** Model Performance Summary on the Held-Out Test Set ROC, receiver operating characteristic; AUC, area under the curve.

Metric	Value
Test accuracy	~89%
ROC AUC	~0.96
F1-Score	~0.88
Precision (Autism class)	~0.89
Recall (Autism class)	~0.87
Training epochs to convergence	~40
Architecture	ResNet-50 (transfer learning)

The training process was stable. Both training and validation loss decreased smoothly without any unusual behavior. The ROC curve shows strong separation between the two classes, which means the model is able to distinguish well between autism and non-autism cases.

A slight imbalance in the confusion matrix was observed. The model performs slightly better on the non-autism class compared to the autism class, but the difference is very insignificant.

We also explored the learned feature representations using t-SNE (T-distributed Stochastic Neighbor Embedding) and UMAP (Uniform Manifold Approximation and Projection) visualizations. The projected embeddings showed observable clustering trends between the two classes; however, these visualizations are intended only as qualitative exploratory tools and should not be interpreted as confirmatory evidence of model generalization or clinical robustness.

For privacy reasons, we did not include any image-based visual outputs in this version. Instead, all results are summarized using metrics and analysis.

## Discussion

Hopefully, it is clear that our main intention behind building this system is to assist clinicians and help organize the screening workflow in a more structured way. We do not claim that the proposed framework can solve all ASD screening-related challenges, nor is it intended to replace clinicians or clinical diagnosis.

The current framework mainly focuses on collecting observations from caregivers, parents, or schools over a longer period of time and converting them into a structured format that clinicians may use during assessment [21-23]. The system is also designed to withhold outputs when confidence conditions are not satisfied [24,25]. At the same time, real-world clinical behavior of the framework still requires further validation using clinically verified datasets and clinician-guided studies.

Another design decision that is worth mentioning is that we wanted to include the core essence of Agentic AI, and that is the reason why we have separated the entire workflow into multiple specialized components, and each component should be handled by a separate specialized agent. Responsibilities such as consent validation, bias monitoring, confidence evaluation, and reporting were intentionally separated so that governance rules can be monitored, audited, and maintained more easily over time.

The current Stage 1 evaluation mainly validates governance behavior and controlled model performance rather than real-world clinical effectiveness. The governance testing was performed using synthetic scenarios under controlled conditions to evaluate policy handling, abstention behavior, escalation flow, and auditability. Separately, the image-based ResNet-50 benchmark achieved around 89% accuracy and an area under the curve (AUC) of about 0.96 on a held-out public test dataset. These two evaluations represent different parts of the framework and should not be interpreted as full clinical validation of the overall system.

Although the current results are promising from a proof-of-concept perspective, this article should be considered as the beginning of building a broad system for ASD screening. In this phase, we are able to conceptualize the framework, and all the broader validation using clinician-verified datasets, stress testing, and clinician-guided evaluation will be performed in the subsequent phases.

Limitations and future work

Although the proposed framework shows promising early results, the current work still has several important limitations.

First, the current study is mainly conceptual, where all the validations were performed in a controlled environment in which we validated governance rules and trained only one model using publicly available facial data. So, it should be considered as a preliminary proof-of-concept result rather than full clinical validation.

Second, the dataset doesn't contain various demographic metadata. So, with this dataset, it is not possible to validate demographic bias [17-20]. In addition, the current study didn't include multimodal validation using audio, video, questionnaires, etc.

Third, the abstention and governance behavior were evaluated in a fully controlled environment. But it is not adequate, and proper validation is required using real-world datasets before we even think of creating a system that can be deployed in real-world settings.

Fourth, although we have conceptualized an agentic AI architecture to assist clinicians in the screening workflow, we have yet to evaluate how this system can be integrated into the existing clinical workflow. So, the usefulness of the generated reports of the system in helping clinicians is yet to be explored.

Overall, in this study, our intention was to conceptualize an agentic AI framework that can be used in the autism screening workflow. We will focus more on real-world validation and testing with multimodal clinically validated datasets in future phases.

## Conclusions

In this article, we tried to envision how agentic AI can eventually help address a real-world healthcare challenge. Based on our prior experience in AI and also our close personal experience with autism, we felt that this is the right time to invest our technological knowledge toward a problem that affects many families and clinicians worldwide.

At the same time, we fully understand that this work is only the first step toward that long-term vision. In this article, we are not claiming that we are going to deliver a full-fledged, clinically certified AI solution; rather, we have explained that we are able to conceptualize a governance-driven framework and perform a preliminary Stage 1 validation under a controlled environment.

We believe that much more work still needs to be done before such systems can become clinically useful and responsibly deployed in real-world settings. In the future phases, we will continue to improve the framework through clinician-guided validation, multimodal datasets, and real-world evaluation until we actually deliver something to our society.

## Appendices

Appendix A: Source code and data availability

Source code: [27]

Dataset source (publicly available): [26]

Appendix B: Synthetic scenario specification

Each scenario in Table 2 specifies (i) an input condition that exercises one or more governance gates; (ii) the expected action per the system policy; (iii) the observed decision issued by the orchestrator; and (iv) the audit signal recorded to the observability layer. Scenarios S-01, S-05, S-06, and S-08 exercise the consent and model-trust gates; S-02, S-04, S-07, and S-10 exercise the confidence and applicability gates; S-03 and S-09 exercise the end-to-end happy path including the caregiver-language vocabulary filter.

Appendix C: LangGraph

LangGraph is a framework for building stateful, multi-step AI workflows using graphs, where nodes represent tasks (e.g., agents, tools, models) and edges define how control and data flow between them-enabling structured, traceable, and controllable agent orchestration (https://www.langchain.com/langgraph).

Appendix D: Bias detection

It checks whether the system treats some inputs differently from others. While the proposed framework is multimodal, the current Stage 1 implementation is trained only on image data. In this setup, we monitor how confident the system is and how often it makes mistakes across different sets of images.

For example, if the system is very confident for some images but often unsure or incorrect for others, this may indicate potential bias. In such cases, the system does not rely on the result but instead sends it for human review to ensure fairness and safety. Future work will extend this approach to additional modalities such as audio, video, and text.

Appendix E: Detailed information about the dataset  

The image dataset used in this study was obtained from the publicly available “Autistic Children Facial Image Dataset” on Kaggle [26]. The dataset contains 2,926 RGB JPEG facial images of children and is already divided into training (n = 2,526; 1,263 autistic and 1,263 non-autistic), validation (n = 200; 100 per class), and test (n = 200; 100 per class) sets. The dataset is class-balanced across all splits. The original image resolutions differ widely, which reflects different real-world image capture conditions. To maintain uniformity during training, all images were resized to 224 × 224 pixels and normalized using standard ImageNet channel-wise mean and standard deviation values. During training, data augmentation techniques such as random horizontal flips, small rotations, color jitter, and affine translations were applied to improve generalization. Validation and test images only underwent deterministic resizing and normalization.

The proposed model was developed using a ResNet-50 backbone pre-trained on ImageNet. Most backbone layers were frozen during training, while the final two residual blocks (layer3 and layer4) were fine-tuned to adapt the model to the ASD classification task. The original classification head was replaced with a custom, fully connected architecture consisting of multiple linear layers, batch normalization, ReLU activation, and dropout layers for regularization. Training was conducted for up to 20 epochs with a batch size of 32 using the AdamW optimizer and a learning rate of 1 × 10⁻⁴. A cosine annealing scheduler was used to gradually reduce the learning rate during training. Cross-entropy loss with label smoothing was used as the objective function. Early stopping monitored validation accuracy and restored the best-performing model weights to reduce overfitting.

The dataset does not provide structured demographic information. The images only contain numeric filenames and binary labels (autistic or non-autistic), without details such as age, gender, ethnicity, geographic origin, or how the diagnosis was clinically confirmed. The original dataset authors also did not clearly mention how the labeling was performed. Because of this, it becomes difficult to measure subgroup representation, demographic balance, or diagnostic reliability using standardized clinical tools such as ADOS-2 or ADI-R.

At the same time, the dataset naturally contains variations in image quality, facial expression, head pose, and capture conditions. In one way, this variability may help the model become more adaptable to practical real-world situations. However, it can also introduce unwanted noise that is unrelated to ASD classification. So, the findings should be interpreted carefully, as the dataset may not fully represent broader clinical populations.

It is very important to note that facial-image-based prediction alone should not be treated as a clinical diagnostic system, as multiple characteristics such as behavioral observations, developmental history, sensory information, and standardized clinical assessments are required to determine whether a child is on the ASD spectrum or not. In our work, facial-image analysis is used only as a single-modality proof of concept within a larger multimodal governance-driven framework. Real-world deployment would still require additional modalities such as video, audio, questionnaires, and text, along with validation on larger and clinically verified datasets.

Appendix F: Confidence thresholds

Confidence thresholds are treated as configurable parameters rather than fixed values, and are intended to be calibrated based on model performance and deployment context. We will also provide a clearer description of how confidence is evaluated (e.g., prediction probability and signal consistency) and how abstention decisions are triggered.

Appendix G: Stage 1 validation

Stage 1 validation refers to a controlled, simulation-based evaluation phase focused on verifying the correctness of the proposed governance-driven agentic framework. This includes validating key system behaviors such as consent enforcement, input applicability checks, bias monitoring, confidence-based abstention, and auditability under predefined test scenarios.

At this stage, the objective is not real-world clinical deployment or large-scale model performance evaluation, but to ensure that the core governance logic and agent orchestration function as intended in a controlled environment.

Subsequent stages will involve expanded validation using real-world datasets, multimodal inputs, and clinician-in-the-loop evaluation.

Appendix H: Test cases 

The GitHub repository contains governance workflow simulations, synthetic test-event definitions, orchestration logic, and representative policy validation cases used during Stage 1 evaluation.

https://github.com/dbashis31/autism-screening-app/blob/main/README.md

Appendix I:  Formal specifications of each agent's flowchart and algorithms 

https://github.com/dbashis31/autism-screening-app/blob/main/algorithm1_flowchart.png 

https://github.com/dbashis31/autism-screening-app/blob/main/algorithm2_flowchart.png

https://github.com/dbashis31/autism-screening-app/blob/main/algorithm3_flowchart.png

https://github.com/dbashis31/autism-screening-app/blob/main/algorithm4_flowchart.png

https://github.com/dbashis31/autism-screening-app/blob/main/algorithm5_flowchart.png 

Appendix J: Fail-Safe mechanism

A fail-safe mechanism is one in which the system does not generate output if critical checks fail. For example, if input validation is incomplete, logs are missing, or model traceability cannot be verified, the system abstains from producing a result and escalates the case for human review. This ensures that no decision is made without proper validation, auditability, and oversight.
